# Supplementary material for: Simple and Fast Prediction of Gestational Diabetes Mellitus Based on Machine Learning and Near-Infrared Spectra of Serum: A Proof of Concept Study at Different Stages of Pregnancy
Source: Biomedicines. 2024 May 21;12(6):1142. doi: 10.3390/biomedicines12061142 (PMC11200648; doi:10.3390/biomedicines12061142)
Supplement: Supplementary file 1 [file biomedicines-12-01142-s001.zip › Supplementary material - Tables.pdf]

Supplementary Material

Table S1. Predictive performance of ML models using NIR spectral data from first trimester serum samples (Full, 10500-4000 cm^{-1}).

Model number	Dataset	Detail	Pretreatment	Width	Sp (Av)	Sp (StD)	Se (Av)	Se (StD)	NER (Av)	NER (StD)
1	NIR1T	Full	MC		0.6185	0.0459	0.3253	0.1091	0.4719	0.0592
2			N+MC		0.6490	0.0445	0.4347	0.0886	0.5418	0.0496
3			WLS+MC		0.7143	0.0372	0.3013	0.0906	0.5078	0.0490
4			WLS+N+MC		0.6970	0.0314	0.3093	0.0891	0.5032	0.0472
5			SNV + MC		0.6749	0.0396	0.3493	0.0948	0.5121	0.0514
6			SNV + WLS + MC		0.7000	0.0433	0.1627	0.0810	0.4313	0.0459
7			SNV + N + MC		0.6803	0.0448	0.3333	0.0808	0.5068	0.0462
8			SNV + WLS + N + MC		0.6970	0.0314	0.3093	0.0891	0.5032	0.0472
9			1D + MC	3	0.8212	0.0262	0.0827	0.0370	0.4519	0.0227
10				7	0.8140	0.0222	0.1080	0.0444	0.4610	0.0248
11				11	0.8006	0.0326	0.1213	0.0440	0.4610	0.0274
12				15	0.7266	0.0317	0.1760	0.0657	0.4513	0.0364
13				19	0.7036	0.0471	0.1787	0.0767	0.4411	0.0450
14				23	0.6519	0.0528	0.2133	0.0738	0.4326	0.0454
15			1D + N + MC	3	0.8239	0.0268	0.0720	0.0183	0.4479	0.0162
16				7	0.8236	0.0287	0.1120	0.0475	0.4678	0.0278
17				11	0.7824	0.0333	0.1027	0.0430	0.4425	0.0272
18				15	0.7481	0.0310	0.1640	0.0649	0.4560	0.0360
19				19	0.7296	0.0404	0.1680	0.0777	0.4488	0.0438

20		23	0.6782	0.0529	0.1960	0.0743	0.4371	0.0456
21	2D + MC	3	0.7854	0.0263	0.1147	0.0405	0.4500	0.0241
22		7	0.7821	0.0252	0.0400	0.0426	0.4110	0.0248
23		11	0.8027	0.0277	0.1000	0.0431	0.4513	0.0256
24		15	0.8131	0.0294	0.1440	0.0492	0.4786	0.0287
25		19	0.7463	0.0321	0.1347	0.0436	0.4405	0.0271
26		23	0.7579	0.0291	0.1213	0.0613	0.4396	0.0339
27	2D + N + MC	3	0.7860	0.0296	0.1040	0.0385	0.4450	0.0243
28		7	0.7737	0.0352	0.0333	0.0387	0.4035	0.0262
29		11	0.8227	0.0290	0.1160	0.0568	0.4693	0.0319
30		15	0.8224	0.0194	0.1560	0.0439	0.4892	0.0240
31		19	0.7484	0.0302	0.1293	0.0283	0.4388	0.0207
32		23	0.7582	0.0316	0.1307	0.0485	0.4444	0.0289
33	SM + MC	3	0.6158	0.0449	0.3267	0.0964	0.4712	0.0532
34		7	0.6143	0.0430	0.3400	0.0992	0.4772	0.0541
35		11	0.6131	0.0462	0.3360	0.1017	0.4746	0.0558
36		15	0.6322	0.0448	0.2880	0.1154	0.4601	0.0619
37		19	0.6278	0.0454	0.2960	0.0925	0.4619	0.0515
38		23	0.6239	0.0522	0.3000	0.0955	0.4619	0.0544
39	SM + SNV + MC	3	0.6934	0.0454	0.3453	0.0669	0.5194	0.0404
40		7	0.6946	0.0503	0.3187	0.0789	0.5066	0.0468
41		11	0.6964	0.0404	0.3320	0.0731	0.5142	0.0418
42		15	0.7179	0.0477	0.3373	0.0692	0.5276	0.0420
43		19	0.7197	0.0504	0.3480	0.0811	0.5339	0.0478
44		23	0.7072	0.0529	0.3387	0.0784	0.5229	0.0473
45	SM + SNV + WLS + MC	3	0.7012	0.0554	0.1667	0.0635	0.4339	0.0421
46		7	0.7322	0.0435	0.2000	0.0700	0.4661	0.0412
47		11	0.7439	0.0485	0.1947	0.0806	0.4693	0.0470
48		15	0.7528	0.0411	0.2453	0.0824	0.4991	0.0461
49		19	0.7540	0.0409	0.2373	0.0605	0.4957	0.0365

50		23	0.7645	0.0421	0.2227	0.0733	0.4936	0.0423
51	SM + SNV + N + MC	3	0.6815	0.0458	0.3307	0.0785	0.5061	0.0454
52		7	0.7060	0.0551	0.3387	0.0748	0.5223	0.0465
53		11	0.7051	0.0524	0.3293	0.0779	0.5172	0.0469
54		15	0.7030	0.0461	0.3480	0.0865	0.5255	0.0490
55		19	0.7101	0.0507	0.3520	0.0700	0.5311	0.0432
56		23	0.7248	0.0468	0.3400	0.0777	0.5324	0.0453
57	SM + SNV + WLS + N + MC	3	0.6934	0.0386	0.2973	0.0906	0.4954	0.0492
58		7	0.6958	0.0382	0.3027	0.1001	0.4992	0.0536
59		11	0.6964	0.0385	0.2853	0.0884	0.4909	0.0482
60		15	0.7099	0.0422	0.2573	0.0863	0.4836	0.0480
61		19	0.7152	0.0420	0.2600	0.0945	0.4876	0.0517
62		23	0.7119	0.0416	0.2760	0.0863	0.4940	0.0479
63	SM + WLS + MC	3	0.7131	0.0391	0.3200	0.0774	0.5166	0.0433
64		7	0.7200	0.0347	0.2947	0.0904	0.5073	0.0484
65		11	0.7164	0.0378	0.2853	0.0894	0.5009	0.0485
66		15	0.7182	0.0433	0.2533	0.0893	0.4858	0.0497
67		19	0.7322	0.0491	0.2493	0.0979	0.4908	0.0548
68		23	0.7278	0.0445	0.2573	0.0785	0.4925	0.0451
69	SM + WLS + N + MC	3	0.6976	0.0408	0.3227	0.1038	0.5101	0.0557
70		7	0.6875	0.0439	0.3093	0.0997	0.4984	0.0545
71		11	0.6967	0.0343	0.3160	0.0795	0.5064	0.0433
72		15	0.7122	0.0421	0.2680	0.0791	0.4901	0.0448
73		19	0.7158	0.0432	0.2733	0.0875	0.4946	0.0488
74		23	0.7045	0.0412	0.2773	0.0705	0.4909	0.0408
75	SM + N + MC	3	0.6451	0.0408	0.4440	0.0826	0.5445	0.0461
76		7	0.6681	0.0488	0.4333	0.0875	0.5507	0.0501
77		11	0.6824	0.0443	0.4427	0.0747	0.5625	0.0434
78		15	0.6687	0.0559	0.4347	0.0765	0.5517	0.0474
79		19	0.6955	0.0461	0.4440	0.0733	0.5698	0.0433

80	23	0.6946	0.0456	0.4507	0.0681	0.5726	0.0410

NIR: near-infrared; 1T: first trimester; Sp: specificity; Se: sensitivity; NER: non-error rate; Av: average; StD: standard deviation; MC: mean centering; N: normalization; WLS: weighted least squares; SNV: standard normal variate; 1D: first derivative; 2D: second derivative; SM: smoothing.

Table S2. Predictive performance of ML models using NIR spectral data from first trimester serum samples (range 1, 10500-7600 cm^{-1}).

Model number	Dataset	Detail	Pretreatment	Width	Sp (Av)	Sp (StD)	Se (Av)	Se (StD)	NER (Av)	NER (StD)
1	NIR1T	R1	MC		0.6245	0.0328	0.4320	0.1010	0.5282	0.0531
2			N+MC		0.6722	0.0361	0.5920	0.0910	0.6321	0.0489
3			WLS+MC		0.7373	0.0549	0.1480	0.0753	0.4427	0.0466
4			WLS+N+MC		0.7499	0.0395	0.3067	0.0883	0.5283	0.0484
5			SNV + MC		0.6800	0.0406	0.4133	0.0738	0.5467	0.0421
6			SNV + WLS + MC		0.7579	0.0458	0.1320	0.0768	0.4450	0.0447
7			SNV + N + MC		0.6764	0.0346	0.4120	0.0793	0.5442	0.0433
8			SNV + WLS + N + MC		0.7433	0.0325	0.3840	0.0732	0.5636	0.0401
9			1D + MC	3	0.8146	0.0272	0.0987	0.0387	0.4566	0.0236
10				7	0.8239	0.0289	0.1147	0.0382	0.4693	0.0240
11				11	0.7857	0.0306	0.1240	0.0426	0.4548	0.0262
12				15	0.7203	0.0274	0.1987	0.0579	0.4595	0.0320
13				19	0.7110	0.0437	0.2120	0.0816	0.4615	0.0463
14				23	0.6555	0.0543	0.2227	0.0837	0.4391	0.0499
15			1D + N + MC	3	0.8358	0.0273	0.0907	0.0399	0.4632	0.0242
16				7	0.8352	0.0226	0.1160	0.0400	0.4756	0.0230
17				11	0.7839	0.0282	0.1267	0.0387	0.4553	0.0239
18				15	0.7439	0.0355	0.2093	0.0646	0.4766	0.0369
19				19	0.7269	0.0372	0.2067	0.0765	0.4668	0.0425
20				23	0.6493	0.0518	0.2253	0.0736	0.4373	0.0450
21			2D + MC	3	0.7985	0.0214	0.1173	0.0394	0.4579	0.0224
22				7	0.7761	0.0305	0.0653	0.0369	0.4207	0.0239
23				11	0.8030	0.0250	0.1120	0.0546	0.4575	0.0300

24		15	0.8158	0.0274	0.1520	0.0357	0.4839	0.0225
25		19	0.7403	0.0362	0.1427	0.0426	0.4415	0.0280
26		23	0.7612	0.0342	0.1600	0.0602	0.4606	0.0346
27	2D + N + MC	3	0.7872	0.0252	0.1000	0.0387	0.4436	0.0231
28		7	0.7872	0.0300	0.0453	0.0367	0.4162	0.0237
29		11	0.8257	0.0236	0.1120	0.0475	0.4688	0.0265
30		15	0.8278	0.0270	0.1600	0.0467	0.4939	0.0270
31		19	0.7460	0.0299	0.1333	0.0269	0.4397	0.0201
32		23	0.7627	0.0306	0.1387	0.0536	0.4507	0.0309
33	SM + MC	3	0.6260	0.0423	0.3933	0.0936	0.5097	0.0513
34		7	0.6164	0.0384	0.4147	0.1153	0.5155	0.0608
35		11	0.6301	0.0381	0.3920	0.1132	0.5111	0.0597
36		15	0.6251	0.0314	0.4067	0.0983	0.5159	0.0516
37		19	0.6257	0.0326	0.4213	0.1047	0.5235	0.0548
38		23	0.6218	0.0395	0.3747	0.1142	0.4982	0.0604
39	SM + SNV + MC	3	0.6943	0.0343	0.4253	0.0736	0.5598	0.0406
40		7	0.6851	0.0356	0.4253	0.0818	0.5552	0.0446
41		11	0.6797	0.0388	0.4027	0.0785	0.5412	0.0438
42		15	0.6946	0.0507	0.3653	0.0822	0.5300	0.0483
43		19	0.7000	0.0495	0.3613	0.0842	0.5307	0.0488
44		23	0.7099	0.0460	0.3453	0.0920	0.5276	0.0515
45	SM + SNV + WLS + MC	3	0.7522	0.0577	0.1347	0.0966	0.4435	0.0563
46		7	0.7609	0.0442	0.1640	0.0788	0.4624	0.0452
47		11	0.7779	0.0403	0.1627	0.0740	0.4703	0.0421
48		15	0.7872	0.0405	0.1760	0.0722	0.4816	0.0414
49		19	0.7961	0.0426	0.1893	0.0651	0.4927	0.0389
50		23	0.7878	0.0382	0.2147	0.0811	0.5012	0.0448
51	SM + SNV + N + MC	3	0.6872	0.0395	0.4173	0.0891	0.5522	0.0488
52		7	0.6839	0.0399	0.4160	0.0814	0.5499	0.0453
53		11	0.6890	0.0423	0.3987	0.0731	0.5438	0.0422

54		15	0.7045	0.0426	0.3507	0.0912	0.5276	0.0503
55		19	0.7152	0.0474	0.3427	0.0713	0.5289	0.0428
56		23	0.7087	0.0542	0.3693	0.0764	0.5390	0.0469
57	SM + SNV + WLS + N + MC	3	0.7543	0.0371	0.3613	0.0751	0.5578	0.0419
58		7	0.7469	0.0344	0.3453	0.0870	0.5461	0.0468
59		11	0.7597	0.0405	0.3520	0.1008	0.5559	0.0543
60		15	0.7481	0.0402	0.3253	0.0968	0.5367	0.0524
61		19	0.7522	0.0445	0.2800	0.0873	0.5161	0.0490
62		23	0.7549	0.0385	0.2507	0.0889	0.5028	0.0484
63	SM + WLS + MC	3	0.7475	0.0633	0.1320	0.0731	0.4397	0.0484
64		7	0.7349	0.0725	0.1360	0.0785	0.4355	0.0534
65		11	0.7699	0.0533	0.1347	0.0731	0.4523	0.0452
66		15	0.7678	0.0579	0.1613	0.0809	0.4645	0.0497
67		19	0.8000	0.0431	0.1867	0.0725	0.4933	0.0422
68		23	0.7979	0.0505	0.1747	0.0818	0.4863	0.0481
69	SM + WLS + N + MC	3	0.7522	0.0295	0.3653	0.0606	0.5588	0.0337
70		7	0.7433	0.0361	0.3453	0.0920	0.5443	0.0494
71		11	0.7501	0.0374	0.3387	0.0902	0.5444	0.0488
72		15	0.7561	0.0361	0.3093	0.0911	0.5327	0.0490
73		19	0.7499	0.0410	0.2840	0.0941	0.5169	0.0513
74		23	0.7460	0.0414	0.2813	0.0822	0.5137	0.0460
75	SM + N + MC	3	0.6752	0.0341	0.5653	0.1176	0.6203	0.0612
76		7	0.6663	0.0382	0.5840	0.1048	0.6251	0.0558
77		11	0.6746	0.0415	0.5293	0.0937	0.6020	0.0512
78		15	0.6761	0.0375	0.5480	0.1019	0.6121	0.0543
79		19	0.6842	0.0431	0.5040	0.0935	0.5941	0.0515
80		23	0.6988	0.0455	0.4987	0.0854	0.5987	0.0484

NIR: near-infrared; 1T: first trimester; R1: range 1; Sp: specificity; Se: sensitivity; NER: non-error rate; Av: average; StD: standard deviation; MC: mean centering; N: normalization; WLS: weighted least squares; SNV: standard normal variate; 1D: first derivative; 2D: second derivative; SM: smoothing.

Table S3. Predictive performance of ML models using NIR spectral data from first trimester serum samples (range 2, 7600-5100 cm^{-1}).

Model number	Dataset	Detail	Pretreatment	Width	Sp (Av)	Sp (StD)	Se (Av)	Se (StD)	NER (Av)	NER (StD)
1	NIR1T	R2	MC		0.6063	0.0364	0.5240	0.0819	0.5651	0.0448
2			N+MC		0.5725	0.0312	0.6120	0.1083	0.5923	0.0564
3			WLS+MC		0.6872	0.0402	0.3213	0.0770	0.5042	0.0434
4			WLS+N+MC		0.6675	0.0638	0.1493	0.0780	0.4084	0.0504
5			SNV + MC		0.7313	0.0280	0.4187	0.0674	0.5750	0.0365
6			SNV + WLS + MC		0.6812	0.0424	0.3213	0.0880	0.5013	0.0488
7			SNV + N + MC		0.7293	0.0353	0.4293	0.0752	0.5793	0.0415
8			SNV + WLS + N + MC		0.7015	0.0439	0.3320	0.0994	0.5167	0.0543
9			1D + MC	3	0.6027	0.0403	0.2800	0.1387	0.4413	0.0722
10				7	0.7069	0.0507	0.0920	0.0630	0.3994	0.0404
11				11	0.6666	0.0479	0.1227	0.0651	0.3946	0.0404
12				15	0.6791	0.0377	0.1680	0.0765	0.4236	0.0426
13				19	0.6776	0.0429	0.1800	0.0716	0.4288	0.0417
14				23	0.6812	0.0435	0.1893	0.0801	0.4353	0.0456
15			1D + N + MC	3	0.6188	0.0354	0.3573	0.1304	0.4881	0.0676
16				7	0.6107	0.0650	0.1893	0.0917	0.4000	0.0562
17				11	0.6460	0.0566	0.1387	0.0748	0.3923	0.0469
18				15	0.6430	0.0613	0.1413	0.0682	0.3922	0.0458
19				19	0.6301	0.0456	0.1480	0.0822	0.3891	0.0470
20				23	0.6460	0.0612	0.1853	0.0592	0.4157	0.0426
21			2D + MC	3	0.5331	0.0186	0.6507	0.0825	0.5919	0.0423
22				7	0.6767	0.0597	0.1573	0.0911	0.4170	0.0545
23				11	0.7185	0.0650	0.1093	0.0599	0.4139	0.0442

24		15	0.5722	0.0758	0.1013	0.0833	0.3368	0.0563
25		19	0.6272	0.0664	0.0693	0.0659	0.3482	0.0468
26		23	0.6463	0.0615	0.1093	0.0817	0.3778	0.0511
27	2D + N + MC	3	0.7433	0.0394	0.1840	0.1074	0.4636	0.0572
28		7	0.7006	0.0649	0.1107	0.0654	0.4056	0.0461
29		11	0.6797	0.0552	0.1427	0.0797	0.4112	0.0485
30		15	0.6209	0.0346	0.3027	0.0982	0.4618	0.0521
31		19	0.6391	0.0434	0.2147	0.0974	0.4269	0.0533
32		23	0.6200	0.0659	0.0893	0.0837	0.3547	0.0533
33	SM + MC	3	0.6242	0.0317	0.5253	0.0746	0.5748	0.0405
34		7	0.6134	0.0268	0.5267	0.0854	0.5700	0.0448
35		11	0.6236	0.0277	0.5040	0.0832	0.5638	0.0438
36		15	0.6209	0.0426	0.5333	0.0913	0.5771	0.0504
37		19	0.6191	0.0361	0.5347	0.0878	0.5769	0.0475
38		23	0.6146	0.0310	0.5267	0.0800	0.5706	0.0429
39	SM + SNV + MC	3	0.7218	0.0306	0.4293	0.0689	0.5756	0.0377
40		7	0.7352	0.0327	0.4480	0.0603	0.5916	0.0343
41		11	0.7296	0.0279	0.4440	0.0640	0.5868	0.0349
42		15	0.7158	0.0374	0.4293	0.0787	0.5726	0.0436
43		19	0.7352	0.0283	0.4493	0.0657	0.5923	0.0358
44		23	0.7263	0.0339	0.4533	0.0673	0.5898	0.0377
45	SM + SNV + WLS + MC	3	0.6818	0.0384	0.3440	0.0730	0.5129	0.0412
46		7	0.6731	0.0461	0.2853	0.0831	0.4792	0.0475
47		11	0.6779	0.0485	0.3080	0.0872	0.4930	0.0499
48		15	0.6848	0.0447	0.2880	0.0947	0.4864	0.0524
49		19	0.6854	0.0460	0.3067	0.0893	0.4960	0.0502
50		23	0.6851	0.0488	0.2720	0.0658	0.4785	0.0410
51	SM + SNV + N + MC	3	0.7310	0.0323	0.4213	0.0743	0.5762	0.0405
52		7	0.7310	0.0325	0.4360	0.0663	0.5835	0.0369
53		11	0.7301	0.0313	0.4413	0.0570	0.5857	0.0325

54		15	0.7197	0.0277	0.4440	0.0781	0.5819	0.0414
55		19	0.7230	0.0384	0.4373	0.0648	0.5802	0.0377
56		23	0.7191	0.0337	0.4360	0.0728	0.5776	0.0401
57	SM + SNV + WLS + N + MC	3	0.6937	0.0420	0.3160	0.0931	0.5049	0.0511
58		7	0.6958	0.0395	0.3373	0.0947	0.5166	0.0513
59		11	0.6943	0.0410	0.3133	0.0777	0.5038	0.0439
60		15	0.6949	0.0424	0.3467	0.0883	0.5208	0.0490
61		19	0.6922	0.0387	0.3427	0.0883	0.5175	0.0482
62		23	0.6890	0.0310	0.3827	0.0969	0.5358	0.0509
63	SM + WLS + MC	3	0.6967	0.0331	0.3347	0.0836	0.5157	0.0449
64		7	0.6815	0.0383	0.3373	0.0730	0.5094	0.0412
65		11	0.6878	0.0436	0.3053	0.0884	0.4965	0.0493
66		15	0.6985	0.0402	0.3187	0.0906	0.5086	0.0496
67		19	0.6910	0.0473	0.3160	0.0902	0.5035	0.0509
68		23	0.6928	0.0400	0.3347	0.0857	0.5138	0.0473
69	SM + WLS + N + MC	3	0.7015	0.0399	0.3373	0.0743	0.5194	0.0422
70		7	0.7012	0.0375	0.3227	0.0927	0.5119	0.0500
71		11	0.6982	0.0495	0.3147	0.0797	0.5064	0.0469
72		15	0.6991	0.0361	0.3373	0.0743	0.5182	0.0413
73		19	0.6940	0.0348	0.3387	0.0784	0.5163	0.0429
74		23	0.6913	0.0412	0.3627	0.1079	0.5270	0.0577
75	SM + N + MC	3	0.5678	0.0322	0.6480	0.1035	0.6079	0.0542
76		7	0.5621	0.0406	0.6213	0.0985	0.5917	0.0533
77		11	0.5675	0.0269	0.6320	0.1028	0.5997	0.0531
78		15	0.5612	0.0273	0.6333	0.1071	0.5973	0.0553
79		19	0.5728	0.0330	0.6293	0.0991	0.6011	0.0522
80		23	0.5633	0.0322	0.6373	0.1045	0.6003	0.0547

NIR: near-infrared; 1T: first trimester; R2: range 2; Sp: specificity; Se: sensitivity; NER: non-error rate; Av: average; StD: standard deviation; MC: mean centering; N: normalization; WLS: weighted least squares; SNV: standard normal variate; 1D: first derivative; 2D: second derivative; SM: smoothing.

Table S4. Predictive performance of ML models using NIR spectral data from first trimester serum samples (range 3, 5100-4000 cm^{-1}).

Model number	Dataset	Detail	Pretreatment	Width	Sp (Av)	Sp (StD)	Se (Av)	Se (StD)	NER (Av)	NER (StD)
1	NIR1T	R3	MC		0.5961	0.0295	0.4907	0.0870	0.5434	0.0459
2			N+MC		0.6167	0.0565	0.1387	0.0671	0.3777	0.0439
3			WLS+MC		0.6051	0.0452	0.1627	0.0727	0.3839	0.0428
4			WLS+N+MC		0.6606	0.0307	0.2733	0.0896	0.4670	0.0474
5			SNV + MC		0.6278	0.0351	0.1400	0.0844	0.3839	0.0457
6			SNV + WLS + MC		0.6140	0.0417	0.1200	0.0883	0.3670	0.0488
7			SNV + N + MC		0.6260	0.0407	0.1307	0.0749	0.3783	0.0426
8			SNV + WLS + N + MC		0.6081	0.0519	0.1787	0.0888	0.3934	0.0514
9			1D + MC	3	0.7137	0.0375	0.1147	0.0539	0.4142	0.0328
10				7	0.6394	0.0467	0.1333	0.0797	0.3864	0.0462
11				11	0.6340	0.0407	0.1200	0.0660	0.3770	0.0388
12				15	0.6185	0.0348	0.1240	0.0841	0.3713	0.0455
13				19	0.6310	0.0374	0.1520	0.0884	0.3915	0.0480
14				23	0.6391	0.0401	0.1893	0.0717	0.4142	0.0411
15			1D + N + MC	3	0.7188	0.0339	0.1333	0.0673	0.4261	0.0377
16				7	0.6588	0.0363	0.1400	0.0777	0.3994	0.0429
17				11	0.6439	0.0334	0.1333	0.0785	0.3886	0.0427
18				15	0.6484	0.0413	0.1093	0.0931	0.3788	0.0509
19				19	0.6472	0.0386	0.1747	0.0807	0.4109	0.0447
20				23	0.6337	0.0434	0.1573	0.0828	0.3955	0.0467
21			2D + MC	3	0.7493	0.0261	0.0533	0.0426	0.4013	0.0250
22				7	0.7388	0.0309	0.1240	0.0660	0.4314	0.0364
23				11	0.7445	0.0321	0.1440	0.0412	0.4442	0.0261

24		15	0.7272	0.0353	0.1947	0.0585	0.4609	0.0341
25		19	0.6734	0.0320	0.1600	0.0750	0.4167	0.0408
26		23	0.6672	0.0451	0.1947	0.0658	0.4309	0.0399
27	2D + N + MC	3	0.7591	0.0292	0.0467	0.0431	0.4029	0.0260
28		7	0.7436	0.0264	0.1040	0.0489	0.4238	0.0278
29		11	0.7385	0.0251	0.1453	0.0480	0.4419	0.0271
30		15	0.7104	0.0350	0.1853	0.0729	0.4479	0.0404
31		19	0.6857	0.0320	0.2187	0.0762	0.4522	0.0413
32		23	0.6743	0.0399	0.1947	0.0772	0.4345	0.0434
33	SM + MC	3	0.6018	0.0331	0.4987	0.0677	0.5502	0.0377
34		7	0.5878	0.0340	0.4813	0.0886	0.5345	0.0475
35		11	0.5791	0.0411	0.4773	0.1105	0.5282	0.0590
36		15	0.5866	0.0357	0.5147	0.0809	0.5506	0.0442
37		19	0.5955	0.0358	0.4973	0.0788	0.5464	0.0433
38		23	0.5931	0.0346	0.5133	0.0811	0.5532	0.0441
39	SM + SNV + MC	3	0.6242	0.0356	0.1573	0.0697	0.3908	0.0391
40		7	0.6272	0.0362	0.1600	0.0797	0.3936	0.0438
41		11	0.6272	0.0317	0.1173	0.0720	0.3722	0.0393
42		15	0.6254	0.0377	0.1413	0.0826	0.3834	0.0454
43		19	0.6230	0.0395	0.1347	0.0802	0.3788	0.0447
44		23	0.6251	0.0451	0.1467	0.0841	0.3859	0.0477
45	SM + SNV + WLS + MC	3	0.6233	0.0356	0.1627	0.0854	0.3930	0.0462
46		7	0.6173	0.0441	0.1467	0.0713	0.3820	0.0419
47		11	0.6084	0.0445	0.1627	0.0821	0.3855	0.0467
48		15	0.6242	0.0399	0.1387	0.0671	0.3814	0.0390
49		19	0.6266	0.0437	0.1693	0.0885	0.3980	0.0494
50		23	0.6128	0.0456	0.1893	0.0834	0.4011	0.0475
51	SM + SNV + N + MC	3	0.6322	0.0388	0.1573	0.0722	0.3948	0.0410
52		7	0.6101	0.0462	0.1360	0.0712	0.3731	0.0424
53		11	0.6310	0.0371	0.1427	0.0738	0.3869	0.0413

54		15	0.6072	0.0410	0.1427	0.0725	0.3749	0.0417
55		19	0.6221	0.0447	0.1587	0.0761	0.3904	0.0441
56		23	0.6119	0.0407	0.1440	0.0665	0.3780	0.0390
57	SM + SNV + WLS + N + MC	3	0.6173	0.0453	0.1760	0.0828	0.3967	0.0472
58		7	0.6152	0.0487	0.1853	0.0916	0.4003	0.0519
59		11	0.6051	0.0386	0.1800	0.0677	0.3925	0.0390
60		15	0.6063	0.0422	0.2093	0.0852	0.4078	0.0475
61		19	0.6203	0.0401	0.1827	0.0735	0.4015	0.0419
62		23	0.6164	0.0442	0.1813	0.0884	0.3989	0.0494
63	SM + WLS + MC	3	0.5848	0.0552	0.1493	0.0640	0.3671	0.0422
64		7	0.5958	0.0569	0.1653	0.0788	0.3806	0.0486
65		11	0.6036	0.0414	0.1867	0.0738	0.3951	0.0423
66		15	0.6039	0.0447	0.1880	0.0849	0.3959	0.0480
67		19	0.6131	0.0360	0.1933	0.0777	0.4032	0.0428
68		23	0.5961	0.0405	0.2000	0.0913	0.3981	0.0500
69	SM + WLS + N + MC	3	0.6191	0.0383	0.1600	0.0862	0.3896	0.0472
70		7	0.6176	0.0444	0.1853	0.0833	0.4015	0.0472
71		11	0.6075	0.0436	0.1973	0.0737	0.4024	0.0428
72		15	0.6167	0.0476	0.1920	0.0890	0.4044	0.0505
73		19	0.6182	0.0357	0.1880	0.0900	0.4031	0.0484
74		23	0.6221	0.0301	0.2013	0.0928	0.4117	0.0488
75	SM + N + MC	3	0.6179	0.0464	0.1493	0.0757	0.3836	0.0444
76		7	0.6152	0.0482	0.1560	0.0733	0.3856	0.0439
77		11	0.6134	0.0657	0.1760	0.0860	0.3947	0.0541
78		15	0.6093	0.0540	0.1733	0.0830	0.3913	0.0495
79		19	0.6245	0.0436	0.1440	0.0897	0.3842	0.0499
80		23	0.6078	0.0417	0.1947	0.0970	0.4012	0.0528

NIR: near-infrared; 1T: first trimester; R3: range 3; Sp: specificity; Se: sensitivity; NER: non-error rate; Av: average; StD: standard deviation; MC: mean centering; N: normalization; WLS: weighted least squares; SNV: standard normal variate; 1D: first derivative; 2D: second derivative; SM: smoothing.

Table S5. Predictive performance of ML models combining NIR spectral data of first trimester sera with medical parameters.

NIR 1T range [a]	Plus	Block order [b]	Sp (Av)	Sp (StD)	Se (Av)	Se (StD)	NER (Av)	NER (StD)
Full			0.6946	0.0456	0.4507	0.0681	0.5726	0.0410
Full	Medical	1-2	0.7355	0.0479	0.3147	0.0863	0.5251	0.0493
		2-1	0.7773	0.0432	0.3360	0.0883	0.5567	0.0491
Full simp [c]	Medical simp [c]	1-2	0.7525	0.0564	0.4227	0.1171	0.5876	0.0650
		2-1	0.7728	0.0702	0.4453	0.0947	0.6091	0.0589
R1			0.6722	0.0361	0.5920	0.0910	0.6321	0.0489
R1	Medical	1-2	0.7048	0.0425	0.3827	0.1059	0.5437	0.0570
		2-1	0.7227	0.0483	0.3827	0.0931	0.5527	0.0525
R1 simp [c]	Medical simp [c]	1-2	0.7328	0.0682	0.5027	0.1251	0.6178	0.0712
		2-1	0.7513	0.0560	0.5013	0.1080	0.6263 [d]	0.0608 [d]

[a] Full: 10500-4000 cm^{-1}; R1: 10500-7600 cm^{-1}.

[b] NIR blocks were pretreated using the conditions identified as best in single-block analysis. Medical blocks were pretreated by autoscaling.

[c] Data obtained after variable selection, using the variable importance information from the original equivalent multi-block model.

[d] Best multi-block model. The NIR block consists of 41% of the original R1 variables (591 wavenumbers out of 1450). The medical block consists of 5 variables, history of GDM in a prior pregnancy, family history of DM, BMI, family history of hyperthyroidism and first period age. Its DCV-AUROC is 0.5219 ± 0.0922.

NIR: near-infrared; 1T: first trimester; R1: range 1; Sp: specificity; Se: sensitivity; NER: non-error rate; Av: average; StD: standard deviation.

Table S6. Predictive performance of ML models using NIR spectral data from second trimester serum samples (Full, 10500-4000 cm^{-1}).

Model number	Dataset	Detail	Pretreatment	Width	Sp (Av)	Sp (StD)	Se (Av)	Se (StD)	NER (Av)	NER (StD)
1	NIR2T	Full	MC		0.6528	0.1013	0.0425	0.0598	0.3477	0.0588
2			N+MC		0.6590	0.0645	0.0475	0.0709	0.3532	0.0480
3			WLS+MC		0.5769	0.0498	0.1625	0.1018	0.3697	0.0567
4			WLS+N+MC		0.5210	0.0927	0.1000	0.1211	0.3105	0.0763
5			SNV + MC		0.5738	0.0538	0.2150	0.1337	0.3944	0.0721
6			SNV + WLS + MC		0.5067	0.0868	0.0775	0.1007	0.2921	0.0665
7			SNV + N + MC		0.5728	0.0557	0.1900	0.1191	0.3814	0.0657
8			SNV + WLS + N + MC		0.5210	0.0776	0.0600	0.0808	0.2905	0.0560
9			1D + MC	3	0.7908	0.0317	0.0000	0.0000	0.3954	0.0158
10				7	0.7436	0.0377	0.0400	0.0589	0.3918	0.0350
11				11	0.7651	0.0312	0.0525	0.0623	0.4088	0.0349
12				15	0.7615	0.0329	0.0600	0.0631	0.4108	0.0356
13				19	0.7815	0.0360	0.0675	0.0629	0.4245	0.0362
14				23	0.8149	0.0389	0.0800	0.0606	0.4474	0.0360
15			1D + N + MC	3	0.7974	0.0285	0.0000	0.0000	0.3987	0.0142
16				7	0.7728	0.0284	0.0725	0.0623	0.4227	0.0342
17				11	0.7733	0.0333	0.0900	0.0567	0.4317	0.0329
18				15	0.7708	0.0368	0.0525	0.0623	0.4116	0.0362
19				19	0.7559	0.0388	0.0100	0.0343	0.3829	0.0259
20				23	0.7826	0.0422	0.0450	0.0606	0.4138	0.0369
21			2D + MC	3	0.8677	0.0321	0.0050	0.0247	0.4363	0.0203
22				7	0.7487	0.0284	0.0000	0.0000	0.3744	0.0142
23				11	0.7267	0.0330	0.0000	0.0000	0.3633	0.0165

24		15	0.8036	0.0386	0.0700	0.0676	0.4368	0.0389
25		19	0.7836	0.0328	0.0750	0.0619	0.4293	0.0350
26		23	0.7687	0.0338	0.0425	0.0598	0.4056	0.0343
27	2D + N + MC	3	0.8815	0.0219	0.0000	0.0000	0.4408	0.0110
28		7	0.7559	0.0333	0.0000	0.0000	0.3779	0.0166
29		11	0.7333	0.0332	0.0000	0.0000	0.3667	0.0166
30		15	0.8133	0.0324	0.1150	0.0556	0.4642	0.0321
31		19	0.7887	0.0361	0.0800	0.0606	0.4344	0.0353
32		23	0.7600	0.0376	0.0325	0.0554	0.3963	0.0335
33	SM + MC	3	0.6390	0.0766	0.0425	0.0649	0.3407	0.0502
34		7	0.6415	0.0831	0.0325	0.0554	0.3370	0.0499
35		11	0.6641	0.0694	0.0300	0.0595	0.3471	0.0457
36		15	0.6708	0.0642	0.0450	0.0657	0.3579	0.0459
37		19	0.6846	0.0798	0.0650	0.0808	0.3748	0.0568
38		23	0.6764	0.0667	0.0550	0.0805	0.3657	0.0523
39	SM + SNV + MC	3	0.5718	0.0668	0.2100	0.1055	0.3909	0.0624
40		7	0.5718	0.0467	0.1825	0.1190	0.3771	0.0639
41		11	0.5872	0.0554	0.1475	0.1000	0.3673	0.0572
42		15	0.6000	0.0515	0.1250	0.1101	0.3625	0.0608
43		19	0.6190	0.0720	0.1100	0.1175	0.3645	0.0689
44		23	0.6123	0.0694	0.1500	0.1237	0.3812	0.0709
45	SM + SNV + WLS + MC	3	0.5015	0.0905	0.0700	0.0881	0.2858	0.0631
46		7	0.4903	0.0926	0.0825	0.0897	0.2864	0.0645
47		11	0.5374	0.0804	0.0525	0.0841	0.2950	0.0582
48		15	0.5246	0.0762	0.0675	0.0918	0.2961	0.0596
49		19	0.5472	0.0812	0.0550	0.0721	0.3011	0.0543
50		23	0.5308	0.0887	0.0425	0.0598	0.2866	0.0535
51	SM + SNV + N + MC	3	0.5728	0.0557	0.1900	0.1191	0.3814	0.0657
52		7	0.5713	0.0486	0.1725	0.1208	0.3719	0.0651
53		11	0.5821	0.0513	0.1450	0.1140	0.3635	0.0625

54		15	0.6103	0.0695	0.1300	0.1100	0.3701	0.0650
55		19	0.6164	0.0604	0.1100	0.1119	0.3632	0.0636
56		23	0.6318	0.0706	0.0950	0.0781	0.3634	0.0526
57	SM + SNV + WLS + N + MC	3	0.4995	0.0848	0.0800	0.1003	0.2897	0.0657
58		7	0.5149	0.0766	0.0750	0.0875	0.2949	0.0581
59		11	0.5410	0.1073	0.0550	0.0844	0.2980	0.0683
60		15	0.5072	0.0953	0.0725	0.0841	0.2898	0.0635
61		19	0.5200	0.0770	0.0675	0.0766	0.2938	0.0543
62		23	0.5267	0.0875	0.0850	0.0960	0.3058	0.0649
63	SM + WLS + MC	3	0.5846	0.0647	0.1500	0.1071	0.3673	0.0626
64		7	0.5687	0.0673	0.1575	0.0938	0.3631	0.0577
65		11	0.5959	0.0537	0.1750	0.1101	0.3854	0.0612
66		15	0.5692	0.0667	0.1550	0.0996	0.3621	0.0600
67		19	0.5759	0.0616	0.1400	0.0933	0.3579	0.0559
68		23	0.5769	0.0458	0.1450	0.0923	0.3610	0.0515
69	SM + WLS + N + MC	3	0.5087	0.0820	0.1025	0.0968	0.3056	0.0634
70		7	0.5267	0.1044	0.0925	0.0972	0.3096	0.0713
71		11	0.5359	0.1001	0.0650	0.0919	0.3004	0.0679
72		15	0.5159	0.0860	0.0775	0.0833	0.2967	0.0599
73		19	0.5344	0.0810	0.0850	0.0926	0.3097	0.0615
74		23	0.5190	0.0978	0.0775	0.0974	0.2982	0.0690
75	SM + N + MC	3	0.6508	0.0729	0.0925	0.1066	0.3716	0.0646
76		7	0.6605	0.0808	0.0800	0.0866	0.3703	0.0592
77		11	0.6764	0.0752	0.0750	0.0911	0.3757	0.0591
78		15	0.6974	0.0705	0.0925	0.0904	0.3950	0.0573
79		19	0.7236	0.0744	0.1125	0.0809	0.4180	0.0549
80		23	0.7456	0.0655	0.1125	0.1079	0.4291	0.0631

NIR: near-infrared; 2T: second trimester; Sp: specificity; Se: sensitivity; NER: non-error rate; Av: average; StD: standard deviation; MC: mean centering; N: normalization; WLS: weighted least squares; SNV: standard normal variate; 1D: first derivative; 2D: second derivative; SM: smoothing.

Table S7. Predictive performance of ML models using NIR spectral data from second trimester serum samples (range 1, 10500-7600 cm^{-1}).

Model number	Dataset	Detail	Pretreatment	Width	Sp (Av)	Sp (StD)	Se (Av)	Se (StD)	NER (Av)	NER (StD)
1	NIR2T	R1	MC		0.6682	0.0916	0.0325	0.0609	0.3504	0.0550
2			N+MC		0.6267	0.0714	0.0250	0.0505	0.3258	0.0437
3			WLS+MC		0.7918	0.0451	0.0200	0.0527	0.4059	0.0347
4			WLS+N+MC		0.8754	0.0414	0.1625	0.1218	0.5189	0.0643
5			SNV + MC		0.6805	0.0965	0.0475	0.0663	0.3640	0.0585
6			SNV + WLS + MC		0.6021	0.0711	0.1350	0.1180	0.3685	0.0689
7			SNV + N + MC		0.6503	0.1085	0.0300	0.0539	0.3401	0.0606
8			SNV + WLS + N + MC		0.6862	0.0656	0.1000	0.1071	0.3931	0.0628
9			1D + MC	3	0.7785	0.0365	0.0000	0.0000	0.3892	0.0183
10				7	0.7559	0.0360	0.0375	0.0631	0.3967	0.0363
11				11	0.7646	0.0376	0.0625	0.0631	0.4136	0.0367
12				15	0.7508	0.0324	0.0400	0.0589	0.3954	0.0336
13				19	0.7656	0.0284	0.0675	0.0629	0.4166	0.0345
14				23	0.7795	0.0449	0.0525	0.0623	0.4160	0.0384
15			1D + N + MC	3	0.7928	0.0319	0.0000	0.0000	0.3964	0.0159
16				7	0.7703	0.0362	0.0650	0.0631	0.4176	0.0364
17				11	0.7749	0.0367	0.0900	0.0567	0.4324	0.0338
18				15	0.7533	0.0366	0.0425	0.0598	0.3979	0.0351
19				19	0.7354	0.0465	0.0100	0.0343	0.3727	0.0289
20				23	0.7492	0.0452	0.0300	0.0539	0.3896	0.0352
21			2D + MC	3	0.8103	0.0351	0.0000	0.0000	0.4051	0.0176
22				7	0.7472	0.0302	0.0000	0.0000	0.3736	0.0151
23				11	0.7359	0.0371	0.0025	0.0177	0.3692	0.0205

24		15	0.8236	0.0372	0.0725	0.0672	0.4480	0.0384
25		19	0.7867	0.0295	0.0500	0.0619	0.4183	0.0343
26		23	0.7646	0.0365	0.0625	0.0631	0.4136	0.0365
27	2D + N + MC	3	0.8492	0.0305	0.0000	0.0000	0.4246	0.0152
28		7	0.7533	0.0323	0.0000	0.0000	0.3767	0.0162
29		11	0.7400	0.0384	0.0125	0.0379	0.3763	0.0270
30		15	0.8154	0.0366	0.1125	0.0725	0.4639	0.0406
31		19	0.7867	0.0321	0.0625	0.0680	0.4246	0.0376
32		23	0.7621	0.0298	0.0350	0.0567	0.3985	0.0320
33	SM + MC	3	0.6795	0.0972	0.0150	0.0482	0.3472	0.0543
34		7	0.6821	0.0881	0.0325	0.0554	0.3573	0.0520
35		11	0.6872	0.0777	0.0225	0.0485	0.3548	0.0458
36		15	0.6349	0.0866	0.0225	0.0485	0.3287	0.0496
37		19	0.6395	0.0892	0.0225	0.0485	0.3310	0.0508
38		23	0.6046	0.0902	0.0150	0.0410	0.3098	0.0495
39	SM + SNV + MC	3	0.6744	0.1102	0.0425	0.0598	0.3584	0.0627
40		7	0.6600	0.1153	0.0325	0.0554	0.3463	0.0640
41		11	0.6400	0.0915	0.0300	0.0595	0.3350	0.0546
42		15	0.5985	0.1008	0.0450	0.0747	0.3217	0.0627
43		19	0.5487	0.1031	0.0325	0.0554	0.2906	0.0585
44		23	0.5385	0.1105	0.0150	0.0410	0.2767	0.0589
45	SM + SNV + WLS + MC	3	0.6021	0.0821	0.1250	0.0911	0.3635	0.0613
46		7	0.6087	0.0829	0.1150	0.1207	0.3619	0.0732
47		11	0.5687	0.0764	0.1300	0.1335	0.3494	0.0769
48		15	0.5877	0.0695	0.1775	0.1290	0.3826	0.0733
49		19	0.5836	0.0679	0.2075	0.1303	0.3955	0.0734
50		23	0.5877	0.0647	0.2000	0.1312	0.3938	0.0731
51	SM + SNV + N + MC	3	0.6810	0.1007	0.0400	0.0589	0.3605	0.0583
52		7	0.6595	0.1011	0.0325	0.0609	0.3460	0.0590
53		11	0.6436	0.1146	0.0450	0.0657	0.3443	0.0660

54		15	0.5938	0.1052	0.0400	0.0641	0.3169	0.0616
55		19	0.5595	0.1257	0.0325	0.0609	0.2960	0.0698
56		23	0.5195	0.1116	0.0225	0.0547	0.2710	0.0621
57	SM + SNV + WLS + N + MC	3	0.6764	0.0691	0.1050	0.1082	0.3907	0.0642
58		7	0.6646	0.0669	0.1125	0.1079	0.3886	0.0635
59		11	0.6513	0.0639	0.1550	0.1117	0.4031	0.0643
60		15	0.6169	0.0666	0.1400	0.1303	0.3785	0.0732
61		19	0.6262	0.0521	0.2000	0.1406	0.4131	0.0750
62		23	0.6118	0.0505	0.2725	0.1280	0.4421	0.0688
63	SM + WLS + MC	3	0.8000	0.0541	0.0225	0.0547	0.4113	0.0385
64		7	0.7841	0.0602	0.0250	0.0505	0.4046	0.0393
65		11	0.7785	0.0423	0.0275	0.0523	0.4030	0.0336
66		15	0.7477	0.0664	0.0275	0.0581	0.3876	0.0441
67		19	0.7010	0.0644	0.0250	0.0505	0.3630	0.0409
68		23	0.6472	0.0858	0.0325	0.0706	0.3398	0.0556
69	SM + WLS + N + MC	3	0.6969	0.0659	0.1050	0.1082	0.4010	0.0633
70		7	0.6723	0.0594	0.1000	0.1101	0.3862	0.0625
71		11	0.6349	0.0619	0.1450	0.1021	0.3899	0.0597
72		15	0.6328	0.0585	0.1475	0.0968	0.3902	0.0565
73		19	0.6087	0.0540	0.1925	0.1455	0.4006	0.0776
74		23	0.6067	0.0482	0.1875	0.1218	0.3971	0.0655
75	SM + N + MC	3	0.6123	0.0769	0.0250	0.0505	0.3187	0.0460
76		7	0.6092	0.0933	0.0200	0.0527	0.3146	0.0536
77		11	0.6133	0.0830	0.0450	0.0657	0.3292	0.0529
78		15	0.6108	0.0702	0.0125	0.0379	0.3116	0.0399
79		19	0.5800	0.0910	0.0250	0.0505	0.3025	0.0521
80		23	0.5708	0.0833	0.0250	0.0565	0.2979	0.0503

NIR: near-infrared; 2T: second trimester; R1: range 1; Sp: specificity; Se: sensitivity; NER: non-error rate; Av: average; StD: standard deviation; MC: mean centering; N: normalization; WLS: weighted least squares; SNV: standard normal variate; 1D: first derivative; 2D: second derivative; SM: smoothing.

Table S8. Predictive performance of ML models using NIR spectral data from second trimester serum samples (range 2, 7600-5100 cm^{-1}).

Model number	Dataset	Detail	Pretreatment	Width	Sp (Av)	Sp (StD)	Se (Av)	Se (StD)	NER (Av)	NER (StD)
1	NIR2T	R2	MC		0.6692	0.0602	0.2550	0.1183	0.4621	0.0664
2			N+MC		0.5974	0.0758	0.2900	0.1503	0.4437	0.0842
3			WLS+MC		0.7605	0.0410	0.1325	0.1084	0.4465	0.0579
4			WLS+N+MC		0.8267	0.0426	0.2200	0.0930	0.5233	0.0511
5			SNV + MC		0.6200	0.0540	0.2825	0.1308	0.4513	0.0707
6			SNV + WLS + MC		0.6554	0.0600	0.1550	0.0930	0.4052	0.0553
7			SNV + N + MC		0.6241	0.0527	0.2750	0.1360	0.4496	0.0729
8			SNV + WLS + N + MC		0.7656	0.0577	0.1850	0.0919	0.4753	0.0542
9			1D + MC	3	0.5190	0.0638	0.3175	0.1365	0.4182	0.0753
10				7	0.7477	0.0519	0.1525	0.1109	0.4501	0.0612
11				11	0.7985	0.0466	0.1550	0.1088	0.4767	0.0592
12				15	0.7615	0.0425	0.1650	0.1169	0.4633	0.0622
13				19	0.7574	0.0452	0.1875	0.1049	0.4725	0.0571
14				23	0.7626	0.0528	0.2200	0.1145	0.4913	0.0630
15			1D + N + MC	3	0.6364	0.0592	0.1975	0.1132	0.4170	0.0639
16				7	0.7662	0.0386	0.1550	0.1028	0.4606	0.0549
17				11	0.7892	0.0431	0.2225	0.1080	0.5059	0.0581
18				15	0.7769	0.0352	0.2600	0.1125	0.5185	0.0589
19				19	0.7697	0.0438	0.2550	0.1128	0.5124	0.0605
20				23	0.7595	0.0543	0.2400	0.0869	0.4997	0.0512
21			2D + MC	3	0.5159	0.0314	0.5400	0.1113	0.5279	0.0578
22				7	0.4944	0.0740	0.1975	0.1290	0.3459	0.0744
23				11	0.6605	0.0642	0.1125	0.1108	0.3865	0.0640

24		15	0.6610	0.0688	0.1875	0.1018	0.4243	0.0614
25		19	0.7538	0.0683	0.1400	0.0999	0.4469	0.0605
26		23	0.7595	0.0538	0.1250	0.0911	0.4422	0.0529
27	2D + N + MC	3	0.6821	0.0288	0.3875	0.1191	0.5348	0.0613
28		7	0.7282	0.0489	0.1025	0.0968	0.4154	0.0542
29		11	0.7595	0.0523	0.2050	0.0937	0.4822	0.0536
30		15	0.7282	0.0494	0.1800	0.0950	0.4541	0.0536
31		19	0.7995	0.0442	0.1825	0.0952	0.4910	0.0525
32		23	0.8246	0.0356	0.2025	0.0941	0.5136	0.0503
33	SM + MC	3	0.6554	0.0577	0.1950	0.1105	0.4252	0.0624
34		7	0.6610	0.0519	0.2175	0.1124	0.4393	0.0619
35		11	0.6518	0.0510	0.2450	0.1100	0.4484	0.0606
36		15	0.6349	0.0686	0.2275	0.1000	0.4312	0.0607
37		19	0.6262	0.0536	0.2250	0.1010	0.4256	0.0572
38		23	0.6415	0.0537	0.2275	0.1121	0.4345	0.0621
39	SM + SNV + MC	3	0.6221	0.0581	0.2700	0.1523	0.4460	0.0815
40		7	0.6303	0.0572	0.2750	0.1406	0.4526	0.0759
41		11	0.6359	0.0543	0.2525	0.1394	0.4442	0.0748
42		15	0.6185	0.0550	0.2925	0.1327	0.4555	0.0718
43		19	0.6287	0.0575	0.2775	0.1219	0.4531	0.0674
44		23	0.6323	0.0527	0.2750	0.1237	0.4537	0.0672
45	SM + SNV + WLS + MC	3	0.6374	0.0480	0.1800	0.1076	0.4087	0.0589
46		7	0.6513	0.0579	0.1675	0.1201	0.4094	0.0667
47		11	0.6277	0.0536	0.1525	0.0955	0.3901	0.0548
48		15	0.6395	0.0549	0.1600	0.1131	0.3997	0.0628
49		19	0.6159	0.0711	0.1575	0.1179	0.3867	0.0689
50		23	0.6221	0.0595	0.1550	0.1145	0.3885	0.0645
51	SM + SNV + N + MC	3	0.6159	0.0493	0.2725	0.1255	0.4442	0.0674
52		7	0.6297	0.0635	0.2800	0.1373	0.4549	0.0756
53		11	0.6390	0.0677	0.2825	0.1283	0.4607	0.0725

54		15	0.6287	0.0563	0.2400	0.1402	0.4344	0.0756
55		19	0.6262	0.0595	0.2850	0.1102	0.4556	0.0626
56		23	0.6174	0.0460	0.2550	0.1359	0.4362	0.0717
57	SM + SNV + WLS + N + MC	3	0.7544	0.0518	0.1950	0.0805	0.4747	0.0479
58		7	0.7359	0.0516	0.1675	0.0965	0.4517	0.0547
59		11	0.7328	0.0531	0.1500	0.0875	0.4414	0.0512
60		15	0.7097	0.0476	0.1400	0.0862	0.4249	0.0492
61		19	0.7097	0.0479	0.1625	0.0986	0.4361	0.0548
62		23	0.7159	0.0480	0.2000	0.1185	0.4579	0.0639
63	SM + WLS + MC	3	0.7533	0.0520	0.1425	0.0911	0.4479	0.0525
64		7	0.7523	0.0413	0.1625	0.1018	0.4574	0.0549
65		11	0.7338	0.0480	0.1650	0.0854	0.4494	0.0490
66		15	0.7374	0.0590	0.1375	0.0919	0.4375	0.0546
67		19	0.7251	0.0572	0.1625	0.0809	0.4438	0.0495
68		23	0.7164	0.0612	0.1525	0.0921	0.4345	0.0553
69	SM + WLS + N + MC	3	0.7728	0.0536	0.1875	0.0809	0.4802	0.0485
70		7	0.7477	0.0447	0.1650	0.0854	0.4563	0.0482
71		11	0.7287	0.0568	0.1750	0.0838	0.4519	0.0506
72		15	0.7159	0.0502	0.1375	0.1049	0.4267	0.0581
73		19	0.7154	0.0392	0.1625	0.1136	0.4389	0.0601
74		23	0.7164	0.0561	0.1750	0.1071	0.4457	0.0605
75	SM + N + MC	3	0.6005	0.0558	0.3175	0.1293	0.4590	0.0704
76		7	0.6349	0.0631	0.2775	0.1390	0.4562	0.0763
77		11	0.6015	0.0845	0.3150	0.1389	0.4583	0.0813
78		15	0.5974	0.0692	0.3075	0.1433	0.4525	0.0796
79		19	0.5928	0.0741	0.3025	0.1496	0.4477	0.0835
80		23	0.5928	0.0629	0.3050	0.1560	0.4489	0.0841

NIR: near-infrared; 2T: second trimester; R2: range 2; Sp: specificity; Se: sensitivity; NER: non-error rate; Av: average; StD: standard deviation; MC: mean centering; N: normalization; WLS: weighted least squares; SNV: standard normal variate; 1D: first derivative; 2D: second derivative; SM: smoothing.

Table S9. Predictive performance of ML models using NIR spectral data from second trimester serum samples (range 3, 5100-4000 cm^{-1}).

Model number	Dataset	Detail	Pretreatment	Width	Sp (Av)	Sp (StD)	Se (Av)	Se (StD)	NER (Av)	NER (StD)
1	NIR2T	R3	MC		0.7805	0.0421	0.6375	0.1318	0.7090	0.0692
2			N+MC		0.7626	0.0483	0.4550	0.0937	0.6088	0.0527
3			WLS+MC		0.8108	0.0401	0.5600	0.1217	0.6854	0.0641
4			WLS+N+MC		0.7497	0.0403	0.4425	0.1048	0.5961	0.0561
5			SNV + MC		0.8133	0.0411	0.7075	0.0998	0.7604	0.0540
6			SNV + WLS + MC		0.7913	0.0427	0.6125	0.1049	0.7019	0.0566
7			SNV + N + MC		0.7908	0.0467	0.7025	0.1126	0.7466	0.0610
8			SNV + WLS + N + MC		0.8426	0.0351	0.4925	0.1222	0.6675	0.0636
9			1D + MC	3	0.7215	0.0577	0.1200	0.0944	0.4208	0.0553
10				7	0.7918	0.0465	0.3875	0.1736	0.5896	0.0899
11				11	0.8415	0.0350	0.5925	0.1179	0.7170	0.0615
12				15	0.8713	0.0361	0.7075	0.0783	0.7894	0.0431
13				19	0.7815	0.0526	0.5500	0.1383	0.6658	0.0740
14				23	0.7523	0.0490	0.4975	0.1300	0.6249	0.0695
15			1D + N + MC	3	0.7949	0.0460	0.1600	0.1042	0.4774	0.0570
16				7	0.8082	0.0422	0.3850	0.1533	0.5966	0.0795
17				11	0.8395	0.0451	0.7200	0.1226	0.7797	0.0653
18				15	0.8415	0.0445	0.7200	0.1301	0.7808	0.0688
19				19	0.8026	0.0425	0.7100	0.1113	0.7563	0.0596
20				23	0.7723	0.0403	0.6700	0.1064	0.7212	0.0569
21			2D + MC	3	0.7497	0.0368	0.0000	0.0000	0.3749	0.0184
22				7	0.8431	0.0416	0.0000	0.0000	0.4215	0.0208
23				11	0.6733	0.0553	0.0075	0.0300	0.3404	0.0314

24		15	0.7221	0.0428	0.1625	0.1108	0.4423	0.0594
25		19	0.7446	0.0460	0.1350	0.1067	0.4398	0.0581
26		23	0.7436	0.0455	0.2225	0.0921	0.4830	0.0513
27	2D + N + MC	3	0.7585	0.0302	0.0000	0.0000	0.3792	0.0151
28		7	0.8441	0.0397	0.0100	0.0343	0.4271	0.0262
29		11	0.6923	0.0515	0.0100	0.0343	0.3512	0.0309
30		15	0.7528	0.0394	0.1825	0.1293	0.4677	0.0676
31		19	0.7744	0.0374	0.1350	0.1067	0.4547	0.0565
32		23	0.7641	0.0455	0.2325	0.1237	0.4983	0.0659
33	SM + MC	3	0.7856	0.0442	0.6200	0.1262	0.7028	0.0668
34		7	0.7708	0.0450	0.6750	0.1010	0.7229	0.0553
35		11	0.7605	0.0444	0.6500	0.1129	0.7053	0.0607
36		15	0.7682	0.0433	0.6475	0.0934	0.7079	0.0515
37		19	0.7636	0.0455	0.6400	0.1061	0.7018	0.0577
38		23	0.7759	0.0417	0.6575	0.1095	0.7167	0.0586
39	SM + SNV + MC	3	0.8072	0.0458	0.6925	0.1017	0.7498	0.0558
40		7	0.8015	0.0401	0.6825	0.0952	0.7420	0.0516
41		11	0.8062	0.0472	0.6800	0.1189	0.7431	0.0640
42		15	0.7964	0.0432	0.6950	0.1105	0.7457	0.0593
43		19	0.8103	0.0427	0.6875	0.1108	0.7489	0.0594
44		23	0.8041	0.0477	0.7100	0.1142	0.7571	0.0619
45	SM + SNV + WLS + MC	3	0.7810	0.0337	0.5950	0.1373	0.6880	0.0707
46		7	0.7938	0.0388	0.6425	0.1101	0.7182	0.0584
47		11	0.7862	0.0410	0.6250	0.1010	0.7056	0.0545
48		15	0.7846	0.0405	0.6525	0.1138	0.7186	0.0604
49		19	0.7897	0.0411	0.6475	0.1092	0.7186	0.0583
50		23	0.8010	0.0462	0.6500	0.1041	0.7255	0.0570
51	SM + SNV + N + MC	3	0.8026	0.0360	0.7100	0.0960	0.7563	0.0512
52		7	0.8046	0.0424	0.7075	0.1089	0.7561	0.0584
53		11	0.7954	0.0453	0.7150	0.1042	0.7552	0.0568

54		15	0.7954	0.0416	0.6650	0.1055	0.7302	0.0567
55		19	0.8092	0.0385	0.7125	0.1164	0.7609	0.0613
56		23	0.8164	0.0428	0.6825	0.1217	0.7495	0.0645
57	SM + SNV + WLS + N + MC	3	0.8364	0.0404	0.4975	0.1115	0.6670	0.0593
58		7	0.8436	0.0409	0.5000	0.1010	0.6718	0.0545
59		11	0.8456	0.0326	0.5300	0.1199	0.6878	0.0621
60		15	0.8374	0.0465	0.5100	0.1491	0.6737	0.0781
61		19	0.8282	0.0455	0.5225	0.1399	0.6754	0.0736
62		23	0.8431	0.0432	0.5350	0.1186	0.6890	0.0631
63	SM + WLS + MC	3	0.8010	0.0410	0.5850	0.1223	0.6930	0.0645
64		7	0.8159	0.0488	0.5675	0.1135	0.6917	0.0618
65		11	0.8082	0.0458	0.5650	0.1018	0.6866	0.0558
66		15	0.8159	0.0407	0.5750	0.0945	0.6954	0.0514
67		19	0.8159	0.0436	0.5725	0.1239	0.6942	0.0657
68		23	0.8056	0.0531	0.5825	0.1174	0.6941	0.0644
69	SM + WLS + N + MC	3	0.8328	0.0392	0.4900	0.1207	0.6614	0.0634
70		7	0.8226	0.0550	0.4975	0.0961	0.6600	0.0554
71		11	0.8328	0.0402	0.5150	0.1375	0.6739	0.0716
72		15	0.8405	0.0367	0.5175	0.1042	0.6790	0.0552
73		19	0.8369	0.0318	0.5325	0.1004	0.6847	0.0527
74		23	0.8369	0.0376	0.5300	0.1173	0.6835	0.0616
75	SM + N + MC	3	0.7692	0.0521	0.4475	0.0981	0.6084	0.0555
76		7	0.7692	0.0443	0.4650	0.1158	0.6171	0.0620
77		11	0.7487	0.0508	0.4775	0.1000	0.6131	0.0561
78		15	0.7426	0.0494	0.4850	0.1147	0.6138	0.0625
79		19	0.7508	0.0500	0.4750	0.1101	0.6129	0.0604
80		23	0.7549	0.0565	0.4725	0.1109	0.6137	0.0622

NIR: near-infrared; 2T: second trimester; R3: range 3; Sp: specificity; Se: sensitivity; NER: non-error rate; Av: average; StD: standard deviation; MC: mean centering; N: normalization; WLS: weighted least squares; SNV: standard normal variate; 1D: first derivative; 2D: second derivative; SM: smoothing.

Table S10. Predictive performance of ML models combining NIR spectral data of second trimester sera with medical parameters.

NIR 2T range [a]	Plus	Block order [b]	Sp (Av)	Sp (StD)	Se (Av)	Se (StD)	NER (Av)	NER (StD)
Full			0.8133	0.0324	0.1150	0.0556	0.4642	0.0321
Full	Medical	1-2	0.8718	0.0398	0.3175	0.1048	0.5946	0.0560
Full	Medical	2-1	0.8703	0.0360	0.2950	0.0747	0.5826	0.0415
Full simp [c]	Medical simp [c]	1-2	0.6759	0.0915	0.5125	0.1478	0.5942	0.0869
Full simp [c]	Medical simp [c]	2-1	0.7600	0.0727	0.5275	0.1457	0.6438	0.0814
R3			0.8713	0.0361	0.7075	0.0783	0.7894	0.0431
R3	Medical	1-2	0.8667	0.0319	0.5750	0.1617	0.7208 [d]	0.0824 [d]
R3	Medical	2-1	0.8487	0.0511	0.4875	0.1641	0.6681	0.0860
R3 simp [c]	Medical simp [c]	1-2	0.6492	0.0713	0.4625	0.1562	0.5559	0.0858
R3 simp [c]	Medical simp [c]	2-1	0.7195	0.0748	0.4750	0.1263	0.5972	0.0734

[a] Full: 10500-4000 cm^{-1}; R3: 5100-4000 cm^{-1}.

[b] NIR blocks were pretreated using the conditions identified as best in single-block analysis. Medical blocks were pretreated by autoscaling.

[c] Data obtained after variable selection, using the variable importance information from the original equivalent multi-block model.

[d] Best multi-block model. Its DCV-AUROC is 0.8126 ± 0.0696.

NIR: near-infrared; 2T: second trimester; R3: range 3; Sp: specificity; Se: sensitivity; NER: non-error rate; Av: average; StD: standard deviation.

Table S11. Molecular vibrations and biomolecules that have been associated with the most relevant spectral intervals of the best first trimester model.

Spectral interval	Tentative molecular vibration	Theoretical wavenumber	Potential biomolecule	Reference
10500-9828 cm^{-1}	Second overtone of O-H stretching	9911, 10288-10300 cm^{-1}	Carbohydrates	(32,34)
	Second overtone of N-H stretching	10277-9804 cm^{-1}	Proteins	(32)
8826-7858 cm^{-1}	Second overtone of C-H stretching	8250-8696 cm^{-1}	Carbohydrates and lipids	(32,33)

Table S12. Molecular vibrations and biomolecules that have been associated with the most relevant spectral intervals of the best second trimester model.

Spectral interval	Tentative molecular vibration	Theoretical wavenumber	Potential biomolecule	Reference
5028-4856 cm^{-1}	Combination of N-H stretching + C-N stretching + N-H bending	4925, 4975, 5025 cm^{-1}	Proteins	(32)
	Second overtone of C=O stretching	4926 cm^{-1}	Proteins	(32)
	Combination of N-H stretching + N-H bending	4880-4902 cm^{-1}	Proteins	(32,33)
	Combination of N-H bending + C-N stretching + N-H bending	4878 cm^{-1}	Proteins	(32)
	Combination of N-H stretching + C=O stretching	4866 cm^{-1}	Proteins	(32,33)
	Combination of second overtone of N-H bending + N-H stretching	4854 cm^{-1}	Proteins	(32)
	Combination band of N-H stretching + N-H bending	4850 cm^{-1}	Proteins	(32)
4764-4702 cm^{-1}	Third overtone of C=O-O stretching	4762 cm^{-1}	Carbohydrates	(32)
	Combination of O-H bending + C-O stretching	4762 cm^{-1}	Carbohydrates	(32)
4492-4442 cm^{-1}	Combination bands of C-H	4250-4760 cm^{-1}	Carbohydrates	(32,34)
4392-4364 cm^{-1}	Combination of C-H stretching + CH_2 deformation	4386 cm^{-1}	Carbohydrates	(32)
4302-4268 cm^{-1}	Combination of C-H stretching + CH_2 deformation	4283, 4292 cm^{-1}	Carbohydrates	(32)
4206-4176 cm^{-1}	Combination band of C-H stretching + C-H stretching	4202 cm^{-1}	Lipids	(32)
4096-4000 cm^{-1}	Combination bands of C-H	4049 cm^{-1}	Lipids	(32)
	First overtone of C-N-C stretching	4049 cm^{-1}	Proteins	(32)
	Combination of C-H stretching + C-C stretching + C-O-C stretching	4000 cm^{-1}	Carbohydrates	(32)
